# Factors Affecting Breast Cancer Screening Behavior Among Women in Saudi Arabia: A Retrospective Cross-Sectional Study

**DOI:** 10.7759/cureus.58324

**Published:** 2024-04-15

**Authors:** Samer Alkarak, Ahmed M Badheeb, Ali Al- Dowais, Hessa Alhabes, Khaled Almahwiti, Abdelaziz A Aman, Mana A Alhajlan, Islam Seada, Sarah A Alshamrani, Bassam Alhussein

**Affiliations:** 1 General Surgery, King Khalid University Hospital, Najran, SAU; 2 Oncology, Oncology Center, King Khalid University Hospital, Najran, SAU; 3 E-Health, Eradah Complex Psychiatric & Addiction, King Khalid University Hospital, Najran, SAU; 4 Surgery, King Khalid University Hospital, Najran, SAU; 5 Surgery, Almiqat Hospital, Madinah, SAU; 6 Internal Medicine, King Khalid University Hospital, Najran, SAU; 7 Cardiac Surgery, King Khalid University Hospital, Najran, SAU; 8 Gynecology, Maternity and Children Hospital, Najran, SAU

**Keywords:** breast cancer screening, breast cancer screening barriers, saudi arabia, breast cancer, screening mammogram, breast self-examination

## Abstract

Background

Early detection of breast cancer is crucial for effective treatment and minimizing mortality, requiring effective screening methods like self-examination, clinical examination, and mammography. However, not all women in Saudi Arabia comply with these examinations, and studies examining its practice and barriers of low uptake are scant. The aim of this study is to investigate factors influencing breast cancer screening behavior among women in Saudi Arabia.

Methods

This cross-sectional study involving 806 women from October to November 2022 used an online questionnaire for the data collection process, including questions about demographic characteristics, awareness assessment, breast cancer screening behavior, symptoms, risk factors, and screening programs. Factors affecting the screening behavior were analyzed using the logistic regression model with adjusted odds ratio (AOR) and 95% confidence interval (CI).

Results

Among the 806 women who participated in the study, 479 (59.4%) were under 40 years old, and half of them were urban residents (n = 394, 48.9%). Only 134 subjects (16.6%) had a history of breast screening. Social media (n = 519, 64.5%) was the predominant source of screening information. The primary obstacles to breast cancer screening were the absence of tumor symptoms (n = 333, 41.3%), insufficient knowledge about early detection (n = 249, 31%), lack of time (n = 245, 30%), fear of discovering a tumor (n = 187, 23%), and lack of awareness about screening centers (n = 155, 19%). In regression analysis, predictive factors for breast cancer screening behavior were as follows: age over 40 years old (AOR: 2.56; 95% CI: 1.70-3.87), residents of big cities (AOR: 3.57; 95% CI: 1.02-12.56), positive family history of breast cancer (AOR: 2.53; 95% CI: 1.50-4.28), proximity to the screening center (AOR: 2.56; 95% CI: 1.22-5.39), and using contraceptive pills for more than five years (AOR: 1.78; 95% CI: 1.04-3.04), and were statistically significant (all p-values < 0.05).

Conclusions

In this study, the most perceived barriers to BSE were the absence of tumor symptoms, followed by insufficient knowledge about early detection, lack of time, fear of discovering a tumor, and lack of awareness about screening centers. Additionally, the predictive factors for breast cancer screening behavior were as follows: age over 40 years old, residents of big cities, positive family history of breast cancer, proximity to the screening center, and using contraceptive pills for more than five years. Given the identified factors affecting breast self-examination behavior in this study, public education initiatives are crucial for raising awareness, facilitating self-examination, and ultimately improving health outcomes and reducing breast cancer treatment costs in society.

## Introduction

Breast cancer is a condition in which cells lose control of their natural mechanisms, resulting in abnormal, rapid, and uncontrolled growth in breast tissue [1]. Breast cancer is a major health concern worldwide, and its prevalence and mortality rates vary in different regions [2-4]. In Saudi Arabia, it ranks among the most common and perilous cancers affecting women [5]. Early diagnosis through methods like breast self-examination (BSE) is crucial for improving survival rates and reducing mortality [6]. BSE, being a low-cost, user-friendly, and non-invasive technique, plays a vital role in the early detection of breast cancer [7]. Despite its importance, there is limited research on the prevalence of BSE in Saudi Arabia, revealing insufficient awareness and knowledge about breast cancer among women, including nursing students. For example, a study conducted among female nursing students of Najran University, Najran, Saudi Arabia, showed a low level of BSE among the participants [8]. Another study conducted among adult women in the city of Najran, Saudi Arabia, showed that only 38.2% of the study participants had ever performed BSE [9]. Similarly, a study conducted among women in Jeddah, Saudi Arabia, found that despite positive attitudes toward BSE, this method has performed poorly [10]. Therefore, there is still a need for early detection of breast cancer in Saudi Arabia and to understand the factors affecting BSE. Based on previous studies, there are several factors affecting BSE behavior, including demographic characteristics [11], health education [11,12], awareness and attitude toward breast cancer [9,11,13], protection motivation theory (PMT) [9], health belief model [14], and parental support [13]. Understanding these factors is crucial for designing effective interventions to promote BSE.

According to studies in Saudi Arabia, numerous individuals avoid routine mammography screenings despite existing security measures [15,16]. Bakarman et al. investigated the barriers to using breast cancer screening methods among adult females in Jeddah, Saudi Arabia [14]. The result showed that the most perceived barrier to BSE was women’s concern, while embarrassment and painful procedures were significant barriers to performing mammography and clinical breast examination [14]. As screening programs aim to diagnose diseases before symptoms manifest and potentially reduce breast cancer mortality, this study investigates the factors affecting BSE behavior among women in Saudi Arabia. The aim of this study is to investigate factors influencing breast cancer screening behavior among women in Saudi Arabia. We hope that our findings from the current study will contribute to the wider implementation of breast cancer screening services, alleviating the burden and cost of the disease.

## Materials and methods

This retrospective cross-sectional study was conducted in Najran, Saudi Arabia, from 1 October to 30 November 2022, using an online questionnaire to assess the level of awareness and factors influencing BSE behavior among adult Saudi women. The study was approved by the Ethics Research Committees of King Khalid Hospital (Code: KACST, KSA: H-I1-N-081) in compliance with the ethical standards outlined in the Declaration of Helsinki. Informed consent was obtained from participants, emphasizing voluntary participation, anonymity, and confidentiality. The target population included all women with any educational level and aged 18 and above in Najran, Saudi Arabia. Women with a history of breast cancer were excluded from the study. The data were collected through an online self-test questionnaire that was available on social media. This questionnaire measured the awareness level and BSE behavior of Saudi women for breast cancer warning symptoms, risk factors, and screening programs. The questions included age, marital status, nationality, income level, place of residence, level of education, family history of breast cancer, use of contraceptives, knowledge of screening programs, barriers, and attitudes toward them [15]. The methods currently used for early detection of breast cancer are BSE, breast examination by a midwife or doctor (clinical breast exam), mammography, breast ultrasound, and magnetic resonance imaging (MRI). Also, BSE should be done monthly after the age of 20, with the first week of menstruation being the optimal time. Any swelling, indentation of a part of the skin or nipple, redness, discharge, wound, or skin spots should be considered suspicious.

Sample size

The estimated sample size was 675 participants, determined using the software G-Power, version 3, according to the study objectives and based on previous studies [15,17] regarding the relationship between the variables in the study. The lowest standardized coefficients of β = 0.04 (observing the smallest difference to reach the maximum sample size) and at least 10 variables in the regression model were considered. The confidence level was 95%, and the study power was 80%, with a two-sided assumption.

Statistical analysis

Data were coded and analyzed using IBM SPSS Statistics, version 23.0 (IBM Corp., Armonk, NY). Descriptive statistics were used for qualitative data presentation. Independent samples t-test was used to compare the equality of two means between qualitative variables with the assumption of equality of variances. The chi-square test was used to check the statistically significant relationship between qualitative variables, and Fisher’s exact test was used in case of limitations in the expected frequency. Logistic regression analysis was used to examine separately (raw effects) and simultaneously (comparative effects) variables predicting BSE behavior in women and to show their relationship with each other. Since demographic characteristics and some other factors can affect this relationship, these variables were considered for adjustment in the final analysis. Adjusted odds ratio (AOR) and 95% confidence interval (CI) indicated effect sizes in the model. A significance level of p < 0.05 was considered for all analyses.

## Results

Among the 806 women participating in the study, 661 (82%) were Saudi and 145 (18%) were non-Saudi. The majority (n = 479, 59.4%) were under 40 years old, and most were married (n = 577, 71.6%), followed by single, divorced, and widowed women, including 168 (20.8%), 47 (5.8%), and 14 (1.7%) of the participants, respectively. In all, 578 (71.7%) participants had an average income level, and nearly half were residents of big cities (n = 394, 48.9%). The majority (n = 653, 81%) of participants had no family history of breast cancer in their first-degree relatives (Table 1).

**Table 1:** Demographic characteristics of participants (n = 806)

Demographic characteristics	Subgroups	N (%)
Nationality	Saudi	661 (82.0)
	Non-Saudi	145 (18.0)
Age (years)	<40	479 (59.4)
	≥40	327 (40.6)
Marital status	Single	168 (20.8)
	Married	577 (71.6)
	Widowed	14 (1.7)
	Divorced	47 (5.8)
Income level	Low	123 (15.3)
	Middle	578 (71.7)
	High	105 (13.0)
City ​​of residence	Main city	394 (48.9)
	Small city	370 (45.9)
	Village	42 (5.2)
Family history of breast cancer	Yes	104 (12.9)
	No	653 (81.0)
	Don’t know	49 (6.1)

**Figure 1:**
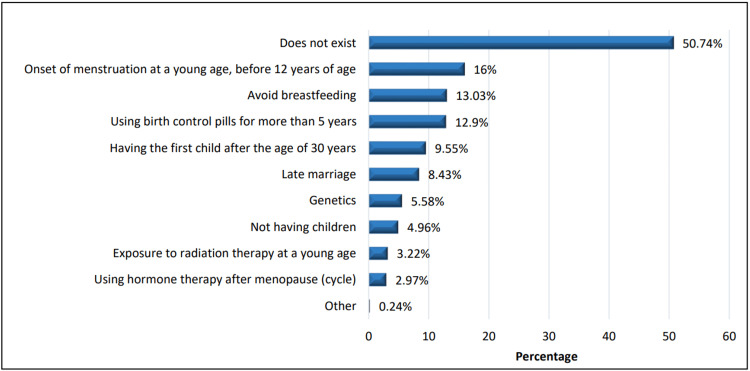
The risk factors associated with BSE among the participants BSE, breast self-examination

Regarding the channels for receiving information about breast cancer, most participants (n = 519, 64.5%) received information about breast cancer through social media (Twitter/YouTube/Telegram, etc.), followed by awareness campaigns (n = 413, 51.2%), TV channels (n = 216, 26.8%), and family/friends (n = 196, 24.3%). Common risk factors included early menstruation (n = 129, 16%), lack of breastfeeding (n = 105, 13.3%), using contraceptive pills for more than five years (n = 104, 12.9%), and having the first child after 30 years of age (n = 77, 9.55%) (Figure 1).

**Figure 2:**
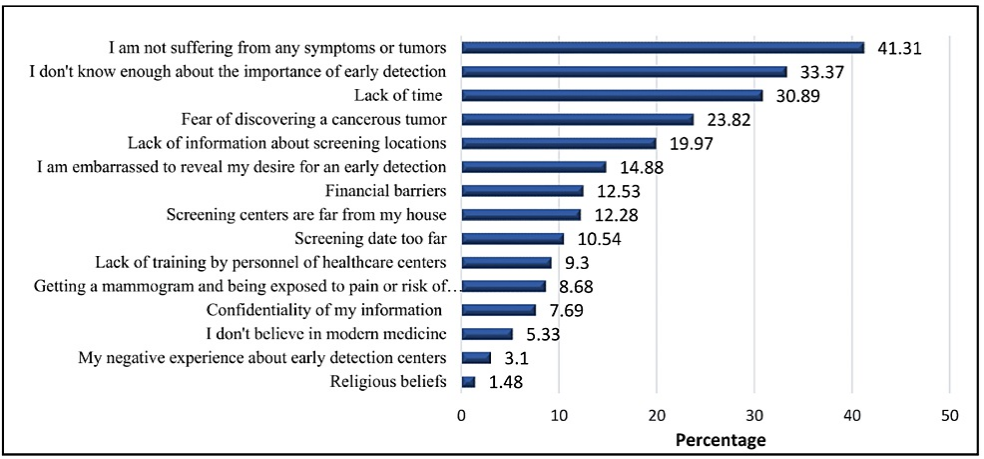
The most important obstacles preventing a woman from performing the BSE BSE, breast self-examination

In this study, the primary obstacles to breast cancer screening were the absence of tumor symptoms (n = 333, 41.3%), insufficient knowledge about early detection (n = 249, 31%), lack of time (n = 245, 30%), fear of discovering a tumor (n = 187, 23%), and lack of awareness about screening centers (19%) (Figure 2).

Only 16.6% of participants (134 individuals) reported a history of breast cancer screening. Other related cases of breast cancer screening are presented in Table 2.

**Table 2:** Knowledge and attitude of BSE among the participants (n = 806) BSE, breast self-examination

Knowledge and attitude of BSE		n (%)
Heard about breast cancer screening	Yes	720 (89.3)
	No	86 (10.7)
How much is known about the importance of early detection?	No knowledge	155 (19.2)
	Intermediate knowledge	386 (47.9)
	Good knowledge	265 (32.9)
Do you think there is enough awareness about breast cancer?	Yes	339 (42.1)
	No	269 (33.4)
	I don’t know	198 (24.6)
Have you ever performed a breast examination?	Yes	134 (16.6)
	No	672 (83.4)
Have you heard about the existence of associations to fight breast cancer?	Yes	438 (54.3)
	No	368 (45.7)
Is there a center/hospital near you that offers early breast cancer screening?	Yes	398 (49.4)
	No	114 (14.1)
	I don’t know	294 (36.5)
Do you trust early breast cancer detection centers and the medical staff who work there?	Yes	490 (60.8)
	No	69 (8.6)
	I don’t know	247 (30.6)
If an electronic program was developed that would facilitate the detection process by maintaining privacy, would you participate in it?	Yes	530 (65.8)
	No	45 (5.6)
	Maybe	231 (28.7)
Would you like to volunteer to raise awareness about the importance of early detection?	Yes	319 (39.6)
	No	210 (26.1)
	Maybe	277 (34.4)

Factors associated with screening behavior:

In regression analysis, predictive factors for breast cancer screening behavior were as follows: age over 40 years old (AOR: 2.56; 95% CI: 1.70-3.87), residents of big cities (AOR: 3.57; 95% CI: 1.02-12.56), positive family history of breast cancer (AOR: 2.53; 95% CI: 1.50-4.28), proximity to the screening center (AOR: 2.56; 95% CI: 1.22-5.39), and using contraceptive pills for more than five years (AOR: 1.78; 95% CI: 1.04-3.04), and were statistically significant (all p-values < 0.05) (Tables 3, 4).

**Table 3:** Binomial logistic regression and crude odds ratios to find the factors associated with BSE BSE, breast self-examination **p-value < 0.05

Variable	Subgroups	B (SE)	Odds ratio (OR)	95% CI of OR	p-value
Nationality	Saudi	Reference group			
	Non-Saudi	0.28 (0.23)	1.32	0.84 to 2.09	0.229
Age (years)	<40	Reference group			
	≥40	1.13 (0.20)	3.08	2.09 to 4.53	0.000**
Marital status	Single	Reference group			
	Married	-0.04 (0.21)	0.96	0.64 to 1.44	0.846
Income level	Low	Reference group			
	Middle	0.55 (0.31)	1.73	0.93 to 3.19	0.081**
	High	0.86 (0.37)	2.37	1.13 to 4.96	0.022
City ​​of residence	Village	Reference group			
	Small city	0.45 (0.62)	1.57	0.46 to 5.33	0.465
	Main city	1.36 (0.61)	3.90	1.17 to 12.93	0.026**
Family history of breast cancer	No	Reference group			
	Yes	0.89 (0.24)	2.44	1.51 to 3.93	0.000**
Heard about BCS	No	Reference group			
	Yes	1.26 (0.47)	3.53	1.40 to 8.90	0.007**
The presence of a nearby BCS center	No	Reference group			
	Yes	1.18 (0.35)	3.26	1.63 to 6.49	0.001**
Trusting BCS centers	No	Reference group			
	Yes	0.61 (0.37)	1.84	0.88 to 3.83	0.103
Late marriage	No	Reference group			
	Yes	0.29 (0.31)	1.33	0.72 to 2.48	0.360
Having the first child after the age of 30 years	No	Reference group			
	Yes	0.39 (0.29)	1.48	0.83 to 2.63	0.179
Not having children	No	Reference group			
	Yes	0.24 (0.41)	1.27	0.57 to 2.82	0.557
Using birth control pills >5 years	No	Reference group			
	Yes	0.67 (0.25)	1.95	1.20 to 3.16	0.007**
Exposure to radiation therapy at an early age	No	Reference group			
	Yes	-0.64 (0.45)	0.53	0.22 to 1.28	0.158
Using hormone therapy after menopause	No	Reference group			
	Yes	0.53 (0.48)	1.70	0.66 to 4.37	0.269

**Table 4:** Binomial logistic regression and adjusted odds ratios to find the factors associated with BSE BSE, breast self-examination **p-value < 0.05 in binomial logistic regression (backward model).

Variable	Subgroups	B (SE)	Adjusted odds ratio (OR)	95% CI of AOR	p-value
Age (years)	<40	Reference group			
	≥40	0.94 (0.21)	2.56	1.70 to 3.87	0.000**
City ​​of residence	Village	Reference group			
	Small city	0.54 (0.65)	1.72	0.47 to 6.17	0.408
	Main city	1.27 (0.64)	3.57	1.02 to 12.56	0.047**
Family history of breast cancer	No	Reference group			
	Yes	0.93 (0.26)	2.53	1.50 to 4.28	0.000**
The presence of a nearby BCS center	No	Reference group			
	Yes	0.94 (0.38)	2.56	1.22 to 5.39	0.013**
Using birth control pills for more than 5 years	No	Reference group			
	Yes	0.57 (0.27)	1.78	1.04 to 3.04	0.035**

## Discussion

This cross-sectional study aimed to investigate the factors influencing BSE behavior in women of Najran, focusing on early breast cancer diagnosis. Several key findings and discussions emerged from the study. Our result showed that the most perceived barriers to BSE were the absence of tumor symptoms, followed by insufficient knowledge about early detection, lack of time, fear of discovering a tumor, and lack of awareness about screening centers. Additionally, the predictive factors for breast cancer screening behavior were age over 40 years old, residents of big cities, positive family history of breast cancer, proximity to the screening center, and using contraceptive pills for more than five years.

The results of the present study reported a low participation rate in breast screening, with only 16.6% of women having a history of breast screening. A previous study from Saudi Arabia has indicated insufficient levels of awareness about breast cancer, leading to inappropriate attitudes and practices toward screening [18]. However, another study in the Western Province of Saudi Arabia showed that most women (98.1%) were aware of breast cancer screening, and 47.7% had already been screened for breast cancer [19]. Another study from Saudi Arabia showed that among 16,000 women invited to a screening program from 2012 to 2019, only 11.9% participated [20]. Therefore, the results show that Saudi women were aware of breast cancer screening, but participation in screening programs was low, which could affect the stage at which breast cancer is diagnosed and potentially affect treatment outcomes. It means that there is a need for consistent, countrywide efforts to enhance breast cancer screening and motivate Saudi women to actively participate in screening programs.

In this study, the absence of tumor symptoms was the main risk factor for neglecting breast cancer screening. Women may believe it is unnecessary since they are not at risk of developing cancer. In Srinath et al. study, among women who have never been screened for breast cancer previously, the most prevalent reason for not engaging in screening activities was a low perceived risk [17]. Additionally, lack of knowledge, not having any symptoms, and being afraid of being diagnosed with breast cancer were the main barriers to practicing BSE among Malaysian women [21]. Furthermore, we found that lack of awareness about the importance of early detection was the second risk factor for neglecting breast cancer screening. Similar to our findings, a study from Indonesia showed that a lack of awareness about the importance of BSE was a barrier to early detection of breast cancer [22]. According to previous reports, women are typically hesitant to have cancer screenings unless their doctor recommends it [17,23]. Guidelines should be streamlined, uniform, and evidence-based to make them easier to recommend. Developing an accurate picture of cancer risk is critical for enhancing the chance that patients will follow medical advice.

Another important impediment to breast cancer screening in this study was the fear of detecting a tumor. Similar to our findings, Alenezi et al. discovered that fear of detecting breast cancer was another important barrier to breast cancer screening in Saudi Arabian women [24]. This information may help target educational programs to improve breast cancer screening.

Given the primary hurdles identified in this study, certain approaches are offered to enhance screening coverage in vulnerable groups. Implementing awareness activities for women in Najran city might help raise their knowledge of the necessity of breast cancer screening. To make initiatives more accessible, they can be launched in frequent venues like schools, media, or supermarkets. Alkhasawneh et al. implemented a breast cancer education program for Arab women, raising awareness and participation in screenings [25]. Serral et al. emphasized the importance of educating the population, especially susceptible women, about the benefits and risks of breast cancer screening programs so that they could make informed decisions [26]. Furthermore, to minimize adherence to screening programs, it is advised that women who often ignore screening get yearly health checkups [27]. Collaborating with healthcare providers is critical for enrolling individuals who satisfy eligibility requirements. Addressing the health information gap and building confidence in healthcare organizations are also critical [27]. Ponce-Blandón et al. highlighted the challenges faced by healthcare workers in handling cultural differences and suggested strategies to educate them about diversity and respect [28]. Ensuring medical confidence in healthcare professionals and systems is crucial for strengthening doctor-patient interactions, increasing satisfaction and adherence to screening, and emphasizing diversity and cultural integration [26-28].

This study identified several significant predictors of BSE behavior among women in Najran, Saudi Arabia, including age, place of residence, family history of breast cancer, proximity to the screening center, using contraceptive pills for more than five years, trusting the health workers, having the first child after the age of 30, and having the required knowledge about BSE screening. These factors align with previous studies, highlighting the influence of age on breast cancer screening behavior. Older women are more likely to do BSE practice [29]. Based on previous research, while the specific city of residence may not directly affect screening behavior, rural or urban residence can be important. For example, the willingness of rural women to participate in breast cancer screening programs was relatively low [30]. As in previous studies, women with a strong family history of this disease are more likely to participate in screening [31,32]. Also, proximity to a screening center can influence screening behavior, especially in rural areas where access to healthcare facilities may be limited [30]. Additionally, awareness about breast cancer screening methods significantly affects screening behavior. Women with more knowledge about screening methods showed a higher participation rate [31]. Other factors addressed in other studies include having complementary health insurance, a history of breast disease, and perceived barriers such as financial problems [33]. Based on the identified predictors, the study recommends targeted interventions to promote breast cancer screening. Public education campaigns should focus on increasing awareness about BSE, regular screening by healthcare professionals, and mammography. Additionally, efforts should be made to increase trust in health workers, potentially through training programs to enhance their knowledge and communication skills related to breast cancer screening. Besides, trying to raise public awareness about breast cancer screening using awareness campaigns in mass media can be very effective.

Our study highlighted that social media platforms, such as Twitter, YouTube, and Telegram, were the primary sources of information about breast cancer screening programs for participants. This aligns with previous research emphasizing the influential role of social media in adolescents’ understanding of BSE [34]. The widespread use of platforms like WhatsApp and Instagram has been shown to enhance knowledge and awareness of BSE. Recognizing social media’s potential, it has become an essential tool for raising awareness and encouraging participation in breast cancer screening programs, self-examination, and mammography, as shown by various studies and analyses [34,35].

The study has several limitations. First, the snowball sampling approach entails a high risk of selection bias. The requirement for participants to recommend people from their social networks may inject a non-random element into the sample, perhaps eliminating persons who are less connected or have different traits. As a result, caution should be taken when extrapolating the findings to the larger population of adult women in Najran or other locations in Saudi Arabia. Second, while various measures were taken to control for social desirability bias, such as ensuring anonymity, conducting the survey online, and using neutral language, it is critical to recognize that complete elimination of this bias is difficult, and self-reported data may still be influenced by it. Third, because the data were collected by self-reporting, there is a risk of reporting bias. Fourth, because the data were cross-sectional, causation cannot be proved, thus use caution when interpreting the findings. Finally, while this study looked at the variables that influence breast cancer screening uptake among adult women in Najran, Saudi Arabia, it is crucial to note that certain critical drivers were not specifically studied. Factors such as the quality of screening services, the competence level of healthcare personnel, cultural perceptions about breast cancer screening, and women’s access to social support were not thoroughly investigated. Future studies should look at these factors to better understand their impact on screening uptake and design focused interventions that address these critical drivers.

## Conclusions

In this study, the most perceived barriers to BSE were the absence of tumor symptoms, followed by insufficient knowledge about early detection, lack of time, fear of discovering a tumor, and lack of awareness about screening centers. Additionally, the predictive factors for breast cancer screening behavior were age over 40 years old, residents of big cities, positive family history of breast cancer, proximity to the screening center, and using contraceptive pills for more than five years. Considering the predictor factors identified in this study for BSE, it is strongly recommended that preventive measures be implemented through comprehensive public education initiatives. These efforts aim to enhance public awareness and facilitate conditions for individuals to engage in BSE, undergo regular screenings by healthcare professionals, and opt for mammography. The establishment of targeted educational plans has the potential to contribute significantly to the reduction of breast cancer prevalence. These measures can lead to improved health outcomes and contribute to the overall reduction in breast cancer treatment costs within the community.
